# Wood-boring beetles associated with *Acacia xanthophloea* in Nairobi and Machakos Counties, Kenya

**DOI:** 10.1371/journal.pone.0188773

**Published:** 2018-03-27

**Authors:** Ruth Kahuthia-Gathu, Duncan Kirubi Thungu, Lucy Wangu, Rachael Kimani

**Affiliations:** Kenyatta University, Department of Agricultural Science and Technology, Nairobi, Kenya; US Department of Agriculture, UNITED STATES

## Abstract

Naivasha thorn tree, *Acacia xanthophloea*, is grown for foliage, timber, shade and rehabilitation of soils in areas with high water tables in Kenya. Its production is threatened by insect pests, which cause major losses. Very little is documented on wood-boring beetles which cause considerable economic damage to lumber used in a variety of applications, and little is known about their natural enemies in Kenya. We conducted the study to evaluate the occurrence of wood-boring beetles on *A*. *xanthophloea* in two different regions of Kenya. Infested wood samples of *A*. *xanthophloea* with fresh exit holes were collected from three sites in Kenyatta University (KU), Nairobi and Mitaboni in Machakos, Kenya. The samples were placed in clear plastic buckets and kept at ambient temperatures 23±2°C, 65±10% relative humidity and 12L: 12D in a laboratory where they were observed daily for adult emergence. Adult beetles were collected every three days for identification and data recording. The experiment was replicated four times and data collected twice a week for 6 months. Data on abundance was subjected to analysis of variance using SAS software. A total of 5,850 and 4,691 beetles were collected where 2,187 and 3,097 were Bostrichidae, accounting for 37% and 66% in KU and Mitaboni, respectively. A total of 12 bostrichid species was identified, including *Sinoxylon ruficorne*, *S*. *doliolum*, *Xylion adustus*, *Xyloperthodes nitidipennis*, *Xyloperthella picea*, *Xylopsocus castanoptera*, *Lyctus brunneus*, *Heterbostrychus brunneus*, *Xylopsocus* sp., and *Dinoderus gabonicus*. The most abundant species in KU was *Xylion adustus* with 1,915 beetles accounting for 88.4%, and *Sinoxylon ruficorne* in Mitaboni with 1,050 beetles accounting for 33.9% of the total. *Sinoxylon ruficorne* was only recorded in Mitaboni while only 2 specimens of *D*. *gabonicus* were found in KU. The mean number of exit holes on *A*. *xanthophloea* differed significantly between sites, which corresponded approximately to the amount of economic damage caused by the beetles to the structural integrity of the lumber. In addition, a number of predators in the family Cerambycidae, Cleridae, Histeridae and parasitoids from Braconidae, Ichneumonidae, and Chalcididae were recovered, suggesting a need to conduct further studies to document these species' diversity, parasitism rates and efficacy for possible biological control.

## Background

*Acacia xanthophloea* belongs to the pod bearing plant family Fabaceae, subfamily Mimosoideae and is commonly known as sulphur bark, Naivasha thorn or fever tree in English, Mgunga in Swahili, and Murera in the Kikuyu dialect of Kenya. There are over 40 species, subspecies and varieties of Acacia represented in Africa. *Acacia xanthophloea* is native to Botswana, Kenya, Malawi, Mozambique, Somalia, South Africa, Swaziland, Tanzania, Zambia, and Zimbabwe [1]. It grows in semi-evergreen bushland and woodland in areas with a high groundwater table near swamps, riverine forests or at lakesides and often forms dense stands in seasonally flooded areas where young branches and leaves are eaten by elephants, leaves and pods by giraffes and velvet monkeys [2]. Other than growing naturally, several species are grown where foliage and pods provide food for livestock during drought [3, 4]. It has also been used in apiculture, as a source of firewood, timber, shade, nitrogen fixing, as live fencing, as nesting sites for birds [1] and for charcoal production especially in areas of its widespread occurrence [5]. Today, the species is also being grown as an ornamental tree. The roots and the bark of stem have been used as medicine against malaria due to their pharmacological properties [1]. *Acacia xanthophloea* has also been used to curb soil erosion and rehabilitation of limestone quarries in Athi River, Kenya [6]. This results in an increase of biodiversity of plants and animals, and in nature restoration. Acacia trees face numerous biotic constraints such as pests and diseases, and abiotic insults from drought or water-logging [7, 8]. Though the vast majority of insects are ecologically important and economically benign, several insect pests attack relatively healthy trees, untreated wood, and downed trees in lumber yards [9]. Some are invasive species that threaten natural forest ecosystems. Plant-insect interactions are quite common in nature and arthropod pests cause direct damage to the plants or their products. Many insects in the family, Bostrichidae, are notorious for locating and infesting freshly cut wood [1, 10]. They are often referred to as powderpost beetles because of their ability to reduce wood to a thin external shell covering the frass produced by the boring activities of the larvae and adults [11]. Generally, Bostrichid beetles prefer to infest dry wood with relatively low moisture content between 8 and 30% [12–13].

Despite the risk of pest introduction from traded agricultural products being manageable, many biological invasions are increasingly recorded worldwide [14]. Some species have been dispersed around the world through timber and timber products trade [11] and many of these invasions are unavoidable due to the pest characteristics [15]. The wood-boring beetles attack the wood at all stages from log to seasoned wood and finished products [16]. They have been recorded in forests, timber depots, sawmills and furniture industries, buildings, seasoned timber, wood artifacts, boxes and packing cases [13]. They obtain nutrition from starch, enabling many species to utilize almost any dry wood material from enormous host range including trees, shrubs, herbaceous plants and bamboo [17– 18]. Most species are polyphagous and attack a wide range of host plants in different families [9]. However, some species are specialist and tend to be confined to a single genus or species of a host plant. The beetles prefer wood with high starch content [19–22], relatively low moisture, or dried wood [23, 24, 22, 13].

Bostrichidae are serious xylophagous pests of trees, forest products, silviculture, agricultural crops and stored vegetable products in most regions of the world, especially in tropical countries [25, 26, 10]. Polyphagous pests inflict greater damage on plants in mixed vegetation systems compared to monophagous pests. Some species have also become pests of stored grain and root crops [27, 10]. However, studies on their ecology are rare and limited information is available. Most of these pests have been introduced accidentally from their native countries possibly via wooden crates or pallets in the cargo shipments. The genus *Sinoxylon* is native to Europe, the Afro-tropical region, the Australian region, the Near East, the Neartic, the Neoptropical region, North Africa and the Oriental region [28, 11] with over 52 species within the genera. It is one the most intercepted species in many countries [29–30]. It is also commonly found in tropical areas, especially in the arid regions, where they damage a wide range of trees [31].

There are two practical usefulness of the study of local species that have unique properties [32]. First, such studies are needed for the protection and use of biodiversity, and secondly for assessment of the impact that may arise due to changes in the environment. Members of some insect families also play an important ecological role in decomposition and nutrient cycling in forest communities [33].

Limited information is available on the insects associated with the multipurpose trees and shrubs that are gaining greater economic importance as components of agroforestry systems. The beetles were chosen because they are the most abundant and diverse group of insects in the environment [34]. In addition, the dynamics of the wood-boring beetles and their natural enemies are governed by the complexity and composition of the agroforestry system. The major aim of this study was to investigate the abundance, species composition, diversity of the wood-boring beetles and their associated natural enemies both predators and parasitoids from *A*. *xanthophloea* growing around Nairobi and Machakos Counties, Kenya.

## Material and methods

### Ethical issues

The research was conducted in the University ground which was part of the daily research encouraged by the University. The second location in Mitaboni, Machakos was conducted in Mr. Matthew Mbuko's farm who invited us for assistance when he saw the trees being affected. This kind of research did not require any granted permission from National Commission for Science and Technology (NACOSTI) nor the University as it does not involve human subjects and interviews.

Secondly, the field studies did not involve any endangered or protected species. *Acacia xanthophloea* grows wild and farmers plant some in some parts of Kenya.

#### Study sites

The survey was conducted in May 2016 from Kenyatta University (KU) and Mitaboni in Nairobi and Machakos Counties, respectively. Kenyatta University (KU) is located 15 Kilometers North of Nairobi at 1°11´ S and 36°55´ E at an altitude of 1,600 meters above sea level. Three sites in KU and Mitaboni where *Acacia xanthophloea* was growing were identified for the studies. The area experiences bimodal rainfall between 1,000–1,400 mm annually with average temperature range of 18–24°C and has clay murram soils. Mitaboni is 17 km North west of Machakos town at 1°21´ S and 37°14´ E at 1,525 m above sea level. The area receives annual rainfall between 600–900 mm, average temperatures of 27°C and comprised of clay soils.

### Abundance of wood-boring beetles in *Acacia xanthophloea*

Purposive sampling was used to collect the dry wood with inactive exit holes, active or freshly bored holes by the wood-boring beetles and those showing damage symptoms [sawdust coming out]. The wood was transversely cut into pieces measuring 15cm long by 10 cm diameter or equivalent volume as described by Sittichaya and Beaver, Kangkamanee [35–36] and placed in clear 20 L plastic containers. The samples were taken to laboratories at KU where they were kept at ambient room temperatures of 23±2°C, 60±10% RH, with a 12 L: 12 D photoperiod. The samples were observed daily and emerging beetles recorded for 8 months. The emerging and positively phototropic adults of the wood-boring beetles were collected every two days, placed in petri dishes and observed under a dissecting microscope. Individual families and species of the wood-boring beetles were identified using morphological and taxonomic characteristics, and their numbers recorded. The Bostrichidae were identified as described by Wisut [37] and Liu et al., [26] while the jewel beetles family Buprestidae by Bellamy [38]. The collected specimens were preserved in 70% ethanol and taken to the National Museums of Kenya (NMK) for confirmation while Bostrichidae were sent to Taiwan for confirmation by Pro. Liu Lan-Yu National Pingtung University, Taiwan.

### Evaluation of number of exit holes of the wood-boring beetles

Five pieces of felled and infested *Acacia xanthophloea* measuring 15cm long by 10 cm diameter collected from three sites in KU and Mitaboni for beetle emergence were used for evaluation of the number of exit holes. Only the number of active exit holes or freshly bored holes by wood-boring beetles was counted from the cut pieces of *A*. *xanthophloea* and recorded.

### Data analysis

The number of beetles was subjected to one way Analysis of Variance (ANOVA) to estimate the mean infestation. When a significant F-value was obtained, the means were compared using the differences of the least square (LS) means at LSMEANS statement, α = 0.05 [39]. The data on abundance was calculated as per cent of the total beetles or organisms collected.

## Results

### Abundance of wood-boring beetles in *Acacia xanthophloea*

Over 5,850 and 4,691 wood-boring beetles were recovered from May 2016 –Feb 2017 from samples of *A*. *xanthophloea* collected in May 2016 at KU and Mitaboni, respectively and kept in the laboratory. Bostrichidae species were the most dominant followed by Curculionidae. Bostrichidae accounted for 2,187 [37.4%] and 3,097 [66%] of the wood-boring beetles in KU and Mitaboni, respectively [Table 1].

**Table 1:** Percent contribution of the family Bostrichidae recovered from *Acacia xanthophloea* at Kenyatta University and Mitaboni.

Study sites	Beetles collected	Bostrichidae	Percent contribution
Kenyatta University	5,850	2,187	37.4
Mitaboni	4,691	3,097	66

A total of 17 and 13 families of the wood-boring beetles, wood-boring beetles in the order Coleoptera and Sub-order Polyphaga were recovered from the sites in Nairobi and Machakos, respectively. The families included Bostrichidae, Buprestidae, Bothridiidae, Curculionidae, Cleridae, Cerambycidae, Chrysomelidae, Ciidae, Colydiidae, Dermestidae, Histeridae, Lyctidae, Tenebrionidae, Staphylinidae, Scolytinae, Silvanidae, Laemophloeidae, and Elateridae [Tables 2 and 3]. Most of the wood-boring beetles were mainly from the Families Bostrichidae, Buprestidae, Curculionidae, Lyctidae and Histeridae. Kenyatta University recorded 16, 6 and 6 species of Bostrichidae, Curculionidae and Lyctidae, respectively. However, 14, 6 and 5 species of the families Bostrichidae, Lyctidae and Curculionidae were obtained from Mitaboni, respectively. At least 16 and 14 species of Bostrichidae wood-boring beetles were recorded from KU and Mitaboni accounting for 51.79% and 73.49%, respectively [Tables 2 and 3]. Other dominant families in KU included Lyctidae, Staphylinidae and Buprestidae [S1 File].

**Table 2:** Wood-boring beetle families recovered from *Acacia xanthophloea* * at Kenyatta University between May 2016–Feb 2017.

Families	No of species	Abundance	Percent [%]
Bostrichidae	16	2,189	51.79
Curculionidae	6	863	20.42
Lyctidae	6	307	7.98
Staphylinidae	2	353	8.35
Buprestidae	4	201	4.76
Cerambycidae	3	127	3.00
Anobiidae	2	109	1.86
Laemophloeidae	2	27	0.64
Dermestidae	2	2	0.04
Silvanidae	1	1	0.02
Scolytidae	1	1	0.02
Bothrideridae	2	16	0.38
Colydiidae	1	23	0.54
Others	4	13	0.31

*The samples were collected in May 2016 and kept in the laboratory for beetle emergence

**Table 3:** Wood-boring beetle families recorded from *Acacia xanthophloea* * in Mitaboni May 2016–Feb 2017.

Families	No of species	Abundance	Percent [%]
**Bostrichidae**	14	3,097	73.49
**Curculionidae**	5	898	21.31
**Lyctidae**	6	135	3.20
**Buprestidae**	1	2	0.05
**Anobiidae**	1	1	0.02
**Cerambycidae**	2	11	0.26
**Dermestidae**	1	33	0.76
**Silvanidae**	1	2	0.05
**Staphylinidae**	3	21	0.5
**Bothrideridae**	1	2	0.05
**Others**	5	5	0.17

*The samples were collected in May 2016 and kept in the laboratory for beetle emergence

Species from the Bostrichidae family included *Sinoxylon ruficorne* Fåharaeus, *S*. *doliolum* Lesne, *Xylion adustus* Fåharaeus, *Xyloperthodes nitidipennis* Murray, *Xyloperthella picea* Oliver, *Xylopsocus castanoptera* Fairmaire, *Lyctus brunneus* Stephens, *Heterbostrychus brunneus* Murray, *Xylopsocus* sp., and *Dinoderus gabonicus* Lesne. The most abundant species in KU were *X*. *adustus* followed by *S*. *doliolum* with 1,915 and 137 beetles accounting for 88.4% and 6.23%, respectively [Table 4]. In Mitaboni, the abundant species were *S*. *ruficorne*, *S*. *doliolum*, *X*. *picea* and X. *nitidipennis* accounting for 33.9%, 19.9%, 15.5 and 10.3%, respectively. *Sinoxylon ruficorne* was only recorded in Mitaboni while *D*. *gabonicus* in KU [S2 File].

**Table 4:** Abundance of Bostrichidae species recorded from *Acacia xanthophloea* * at Kenyatta University and Mitaboni from May 2016 –Feb 2017.

No of species	Kenyatta University		Mitaboni	
	No. beetles	Percent [%]	No. beetles	Percent [%]
***Sinoxylon ruficorne***	-	-	1,050	33.9
***Xylion adustus***	1,915	88.4	108	3.5
***Sinoxylon doliolum***	137	6.23	615	19.9
***Xyloperthella picea***	12	0.3	480	15.5
***Xyloperthodes nitidipennis***	98	2.2	319	10.3
***Xylopsocus castanoptera***	2	0.05	6	0.2
***Lyctus brunneis***	93	1.6	20	0.7
***Dinoderus gabonicus***	-	-	2	0.06

*The samples were collected in May 2016 and kept in the laboratory between May 2016 and Feb 2017 for beetle emergence

Other than the wood-boring beetles, both predators and parasitoids were recovered at Kenyatta University and Mitaboni. The predators included the families Histeridae, Cleridae, Elateridae, Laemopholoidae, Latridiidae, Ciidae, Anthocoridae and Pseudoscorpionida while the parasitoids included Ichneumonidae, Braconidae, Eulophidae, Chalcididae and Pteromalidae which were recorded in both regions. However, Eurytomidae and Eupelmidae were recorded in KU and Mitaboni, respectively.

### Evaluation of number of exit holes of the wood-boring beetles

There was significant difference (df = 5,122; f = 6.32; *P*<0.0001) on the mean number of exit holes made by the wood-boring beetles on the pieces of wood of *A*. *xanthophloea*. It was also observed that samples from Mitaboni had significantly higher number of exit holes compared those recorded at KU [Table 5]. The mean numbers of exit holes was comparable in all the three sites at KU. In Mitaboni, site 2 had the highest number of exit holes while in KU, site 1 had the lowest of 66.4 and 31.1, respectively. The young adults remain tunneling and feeding in the wood for several days before emerging through small circular holes they make in the wood. Most of the pupae and adults were found just below the bark and cause significant damage on the wood reducing it to a powder.

**Table 5:** Mean ±SE number of exit holes of wood-boring beetles on *Acacia xanthophloea* at Kenyatta University and Mitaboni.

Region	Site	N	Number of exit holes
**Kenyatta University**	1	19	31.1±4.7c*
	2	15	39.9±6.1bc
	3	20	32.3±4.9c
**Mitaboni**	1	33	41.7±3.7bc
	2	17	66.4±7.1a
	3	24	59.3±6.7ab

*Means followed by the same letter in the column are not significantly different at *P*≤0.05

## Discussion

A range of wood-boring beetle families were recovered from wood samples of *Acacia xanthophloea* from the selected sites in Nairobi and Machakos Counties, Kenya. These families included Bostrichidae, Buprestidae, Histeridae, Curculionidae, Lyctidae, Chrysomelidae, Dermestidae, Tenebrionidae, Staphylinidae, Cleridae, Cerambycidae, Silvanidae, Laemophloeidae, Ciidae and Elateridae. Similar observations were made [40] who recorded rich beetle communities from *Acacia drepanolobium* in Kenya, which comprised of 13 beetle families mainly Curculionidae, Anthicidae, Cleridae, Buprestidae, Cerambycidae, and Bostrichidae. These results are also comparable with those of Sittichaya et al., 2009 [35] who found high species richness with Bostrichidae being the most abundant and diverse group of insects in the environment in Thailand. The abundance and diversity of the families on the *A*. *xanthophloea* were well adapted to the prevailing climatic and edaphic conditions in both study sites where the temperatures range between 21°C to 30°C [1, 6]. The diverse families and species could be related to high nutritional value from the acacia species [19–22]. Grazers recognize several species of *Acacia* for their feeding value during drought [3, 4]. Sandhu and Fihlo et al., [17–18] reported that the wood-boring beetles obtain nutrition from starch, enabling many species to utilize almost any dry wood material from enormous host range including trees, shrubs, herbaceous plants and bamboo.

The preliminary study shows that wood-boring beetles in the order Coleoptera suborder Polyphaga were the most abundant and diverse group of insects recorded in *A*. *xanthophloea* from both KU and Mitaboni. *Xylion adustus* was the most dominant species at KU accounting for 52.3% of the total beetle recovered during the study. The species has also been reported in Tanzania, Malawi, Southern Africa and South Africa. However, there are no reports regarding their abundance and tree species they infest in Kenya.

Besides Bostrichidae, Buprestidae beetles were recorded with more abundant *Agrilus* species accounting for 5.5% in KU while only 4 specimens were recorded in Mitaboni. Studies conducted by Vladimir and Georgi [41] indicated that the Buprestidae fauna has over 429 species in Kenya. This additional information on Buprestidae adds to the recommendations of Sakalian and Georgi 2013 [42] who also suggested the need for further detailed studies in order to enrich the knowledge on numbers and distribution of the buprestid species in Kenya

It was observed that *S*. *ruficorne* and *S*. *doloilum* were the most abundant species in Mitaboni accounting for 47.8% and 17%, respectively. *Sinoxylon ruficorne* has also been reported on *A*. *mearnsii* in Kenya [43]. However, its abundance was not quantified. The auger beetle *S*. *ruficorne* was also found infesting cassava roots in Mozambique, Nigeria and South Africa while *S*. *doliolum* is distributed in Tanzania [44]. In addition, *X*. *castanoptera* has also been recorded in East Africa, Madagascar and Mauritius. *Dinoderus gabonicus* whose normal distribution is in West Africa was found on *A*. *xanthophloea* at KU [45].

Wood-boring beetles have preference for hardwoods due to presence of pores that enable easy penetration by larvae and laying of eggs by adults. Some of the families infested include Ulmaceae, Euphorbiaceae, Lauraceae, Leguminosae, Dipterocarpaceae, Anacardiaceae, Poaceae, Dipterocarpaceae, Anacardiaceae, and Rubiaceae among others [27, 46, 47]. *Sinoxylon* species are particularly important pests of trees, wood and bamboos [27, 48]. *Sinoxylon conigerum* has been recorded attacking rubber wood *Hevea brasiliensis* Müll., pigeon pea *Cajanus cajan* (L.), mango tree *Mangifera indica* L., cassava stem *Manihot esculenta* Crantz, cotton dry roots *Gossypium hirsutum* L., branches of guava *Psidium guajava* L., and teak (*Tectona*. *grandis*) [49–51]. *Sinoxylon senegalense* is the most notorious pest of the white thorn acacia *Acacia seyal* (Fabaceae *Mimosoideae)* native to Kenya [1]. *Acacia tortilis* is also infested by *S*. *crissum* and auger beetle *S*. *anale*. *Sinoxylon anale* and *S*. *unidentatum* are the most destructive beetles of rubber sawn wood in southern Thailand [52, 26]. *Sinoxylon unidentatum* has also been recorded attacking Ulma-Euphorbiaceae, Lauraceae, Dipterocarpaceae, minosaceaae, Leguminoseae, Anacardiaceae, Rubiaceae [27, 46–47]. *Xylopsocus* species has been recorded in the wood of *Morus alba* L. trunks of “Kimoungoue” in east Africa, bamboo, litchi *Litchi chinensis* Sonn., and grape *Vitis* sp. in Brazil [53, 27]. The beetles are of considerable economic importance to forestry and wood-using industries, especially in tropical countries [26, 11]. The damage is typically caused by the larvae and adults, which bore in the stems, branches, twigs of dead, damaged or stressed host [52, 20, 25–26]. Characteristic signs of infestation include the exit holes produced by the adult beetles which are circular and the diameter depends on the wood-boring species [54], exudation of gummy sap or resin, early branching depending on the infested trees, dieback and death.

The wood-boring beetles caused heavy damage to *A*. *xanthophloea*. This tree has economic value for the local population who utilizes it for timber, firewood, fencing and apiculture. With impending timber shortages and concerns over depleting forest resources in many countries, intense efforts have been made to maximize utilization of forest resources including lesser known or underutilized species, lower grade timbers and also to encourage a more widespread use of sapwood in timber products. Utilization of such materials, which are often of lower resistance to wood degrading insects, has led to greater prominence of *M*. *rugicollis*. *Acacia* has the ability to rapidly absorb nutrients, particularly nitrogen, and incorporate them into biomass after fire, enabling it to act as a pioneer species. This helps to prevent deterioration of the already low-quality soils from the eroding nature of the environment and also helps to conserve nutrients [55–56].

## Conclusion and recommendations

There are numerous families and species diversity of the wood-boring beetles infesting the *A*. *xanthophloea* in Nairobi and Machakos, Kenya. Long distance separating the study sites points to the possibility of a widespread distribution in the country. Since a number of predators and parasitoids families were also recovered in the study, more investigations on host specificity and efficacy of these parasitoids needs to be investigated to determine species that could be used in classical biological control or augmentation in order to keep the wood-boring beetles below economic threshold level.

## Supporting information

S1 File Wood-boring beetle data Kenyatta University. (XLSX)

S2 File Wood-boring beetle data Mitaboni. (XLSX)
